# The Application of Electromagnetic Sensors for Determination of Cherenkov Cone Inside and in the Vicinity of the Detector Volume in Any Environment Known

**DOI:** 10.3390/s21030992

**Published:** 2021-02-02

**Authors:** Valeriu Savu, Mădălin Ion Rusu, Dan Savastru

**Affiliations:** Department of Constructive and Technical Engineering, National Institute of Research and Development for Optoelectronics—INOE 2000, 1 Atomistilor Str., 077125 Măgurele, Romania; savuv@inoe.ro (V.S.); dsavas@inoe.ro (D.S.)

**Keywords:** electromagnetic sensor, Cherenkov cone, environment, neutrinos, electromagnetic wave, cosmic rays

## Abstract

The neutrinos of cosmic radiation, due to interaction with any known medium in which the Cherenkov detector is used, produce energy radiation phenomena in the form of a Cherenkov cone, in very large frequency spectrum. These neutrinos carry with them the information about the phenomena that produced them and by detecting the electromagnetic energies generated by the Cherenkov cone, we can find information about the phenomena that formed in the universe, at a much greater distance, than possibility of actually detection with current technologies. At present, a very high number of sensors for detection electromagnetic energy is required. Thus, some sensors may detect very low energy levels, which can lead to the erroneous determination of the Cherenkov cone, thus leading to information errors. As a novelty, we propose, to use these sensors for determination of the dielectrically permittivity of any known medium in which the Cherenkov detector is used, by preliminary measurements, the subsequent simulation of the data and the reconstruction of the Cherenkov cone, leading to a significant reduction of problems and minimizing the number of sensors, implicitly the cost reductions. At the same time, we offer the possibility of reconstructing the Cherenkov cone outside the detector volume.

## 1. Introduction

Cosmic radiation with energies up to 10^14^ eV can be directly detected by measurements with instruments placed on satellites or balloons. Detection of cosmic radiation with energies higher than 10^14^ eV can be done through indirect experiments. A primary particle with energy over than 10^14^ eV can be detected by determining the effect of the secondary particles that are formed as a result of the interaction of the primary particle (neutrinos) with the respective medium [1,2,3,4].

Some of the cosmic radiation travels a long way to get to the Earth and they pass by AGN (Active Galactic Nuclei), which has the effect of accelerating them. By determining as accurately as possible the spectrum of cosmic radiation can be found information about the mechanisms that acted on their acceleration, the sources that acted on them (in the vicinity of our planet), the phenomena that occurred during propagation, etc. [5]. The analysis of these phenomena is done by the GZK effect (Greisen–Zatsepin–Kuzmin) and represents the theoretical upper limit of cosmic ray energy. The GZK effect provides a threshold of 6 × 10^19^ eV (GZK limit), the energy limit at which, following the interaction of the particles (ultra-high energy cosmic rays (UHECR)) with the background radiation (cosmic microwave background (CMB)) it will lose enough energy such that, cannot be detected above this threshold (particle of the cosmic radiation), if they come from distant sources [6].

The theoretical limit of GZK is applicable to protons emitted by cosmic radiation. In the case of neutrinos, the GZK limit does not apply [7].

Neutrinos with UHE (Ultra High Energy) that interact with environment can have energies of the order (10^15^ ÷ 10^23^) eV. One can easily see the importance of determining the direction from which they come and the energy of neutrinos generated by the phenomena in the universe and carried by cosmic radiation. The Cherenkov cone is generated following the interaction of a neutrino with the environment dens and transparent. The Cherenkov cone (it is a regular cone—from geometrically view; the solid angle at the top of the cone it is preserved) has the height perfectly generated in the extension of the cosmic radiation direction and perpendicular to the side area of cone will generating electromagnetic radiation, which is directly proportional to neutrino energy which generated the Cherenkov cone [8]. The Cherenkov cone has a solid angle at the tip of the cone which represents the measure of the energy of the incident primary particle [9]. The base of the Cherenkov cone moves in the same direction and sense with the direction and the sense of movement of the neutrino that generated the Cherenkov cone.

By using electromagnetic sensors to determine the electromagnetic effect of the Cherenkov cone, which is dependent on the energy and direction of the neutrino generated by the phenomena in the universe, important information for astrophysics and astronomy is discovered. Neutrinos with very high energies can be determined by interacting with any known medium in which a Cherenkov detector is made [10]. Detecting the energy and direction of neutrinos (with very high energies) that interact with the environment in which the Cherenkov detector is placed will bring information about the universe very far away. The information brought by neutrinos with very high energy, is from much greater distances than the possibility detection with current technology, because it does not interact with the environments close to the area where they were generated by phenomena in the universe (their energy being very high, they will pass through the nearby environments without interacting with them; thus, they lose their energy and reaching the Earth with enough energy to interact with the terrestrial environments) and thus, the information obtained will have an important contribution in the knowledge of the universe [11].

So far, the cosmic radiation detectors proposed for transparent and dense dielectric environments use many electromagnetic sensors [12,13], many holes for introduction the sensors and a complicated data processing device, which involve very high costs.

If one or more electromagnetic sensors, do not transmit the data received at the collection point for various reasons (very low energetic level detection by the electromagnetic sensors, significant errors, etc.), then the determination of the Cherenkov cone will be erroneous [14].

In this paper we use reception electromagnetic sensors and aim to eliminate, as far as possible, errors in detecting the Cherenkov cone in environments where it can be achieved by preliminary measurements of dielectric parameters of the environment that can influence the propagation of electromagnetic waves generated by Cherenkov cone and making a spatial map distribution of these parameters. Thus, we can detect the occurrence of errors from a sensor, anticipating the energy level measured by that sensor, because we know the energy levels detected by neighboring sensors and we also know the dielectric parameters of the environment in the vicinity of the sensor that generated the error.

The error minimization method can also be applied in the case of generating the Cherenkov cone outside the volume of the Cerenkov detector. Thus, we aim to determine in the planes outside the Cherenkov detector, where we will determine the location points of the electromagnetic sensors, in order to detect the Cherenkov cone, in case it would form outside the detector volume but inside the space determined by these plans. The realization of this proposal presupposes the fulfillment of an energetic condition. The energy detected by the internal electromagnetic sensors should be higher than the energy of the external ones by the volume of the Cherenkov detector.

### Literature Review

Cosmic radiation was studied more than 100 years ago (1911–1913). Neutrinos of cosmic radiation with energies between 10^15^ ÷ 10^23^ eV, reach the earth. The generation of the Cherenkov cone is given by the interaction of the environment with neutrinos with energies between 10^15^ ÷ 10^21^ eV. Neutrinos with energies higher than 10^21^ eV do not interact with terrestrial environments, they continue their way, further through the universe [15,16,17].

Research on the Cherenkov cone leads to the realization of cosmic radiation observatories in dense and transparent environments for the electromagnetic waves [18]. Cherenkov radiation with coherent radio frequency emission represents, precisely, the Askaryan effect and is given by an excessive electric charge, when an electron cascade develops [19]. The Askaryan effect occurs when particles move through a medium faster than light through that medium [20].

The interaction between a UHE neutrino and a dense and dielectric medium, generates relativistic particles in an avalanche of waterfalls. Only 70% of them are electrically charged generating electromagnetic fields and contributing to the total energy of the waterfall [21,22]. Particles moving faster than light through a dense dielectric medium produce Cherenkov radiation [20].

## 2. Description of the Problem

**Figure 1:**
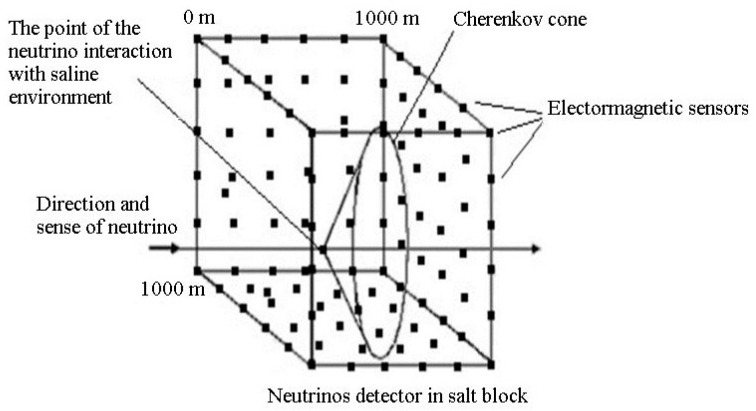
Neutrinos detector in a salt block consisting of electromagnetic sensors placed in a cube with a 1 km side [23].

In order to determine the energy of the electromagnetic waves emitted by the Cherenkov cone in, it is necessary to use wide-band sensors. These sensors will be placed in a cube with side of 1 km (example in Figure 1). In Figure 1 is shown a neutrino detector placed in a salt block consisting of electromagnetic sensors placed in a cube with a side of 1 km [23], according to a recursive rule in plan and space.

Figure 1 shows the use of a very large number of electromagnetic reception sensors for the saline environment. In order to be easier to understand, we will analyze the implications of making a Cherenkov detector in a saline environment. To increase the chances to detect the neutrinos, these will have to cross a mass of matter as consistent and homogeneous as possible. For this, we could use massive blocks of salt deposits as natural detectors (Figure 1). For the detection of ultra-high energy neutrinos (10^23^ eV), it would be useful to choose salt deposits that contain blocks of 3 km × 3 km × 3 km which have masses of the order of 50 Gt [24].

The determination of the number of electromagnetic sensors is given by several factors. The first main factor is given by the geometry of the Cherenkov detector 3D (*x* × *y* × *z*; 500 m × 500 m × 500 m with a 25 m step resulting *n_x_* = *n_y_* = *n_z_* = 500/25 = 20 electromagnetic sensors) [25]. A second factor is given by the number of sub-bands (*n_sb_*) in which the total bandwidth is divided. A third factor is given by the number of the electromagnetic sensors of the same type of polarization (nap) and the last factor is given by the type of polarization, the last being equal to 2. Then it is possible to determine the total number of electromagnetic sensors (*N*):

(1) $$N=\left(n_{x}\times n_{y}\times n_{z}\right)\times n_{sb}\times n_{ap}\times 2$$

So, for a Cherenkov detector with a geometry: (*n_x_* × *n_y_* × *n_z_*) = (20 × 20 × 20), the number of electromagnetic sensors on the axes *x* × *y* × *z*, *n_sb_* = 1 and *n_ap_* = 2; then we will have: *N* = 32,000 electromagnetic sensors and a number (*n_x_* × *n_y_*) = 400 holes in the salt block. If the electromagnetic sensors are narrowband, i.e., approximately 100 MHz and we want to analyze a Cherenkov radiation in a band of 100 MHz ÷ 1 GHz is required, then *n_sb_* = 9; so, we will obtain *N* = 288,000 electromagnetic sensors and a number of (*n_x_* × *n_y_*) = 400 holes in the salt block. It is observed that a more economical option would consist in the use of the electromagnetic wide-band sensors [26].

Following the measurements performed in the salt block at Cantacuzino mine in Slănic Prahova, it was found that there is an optimal frequency for the determination of the electromagnetic radiation generated by the Cherenkov cone produced by the interaction of a high energy neutrino (10^18^ ÷ 10^23^) eV with saline environment. Therefore, a cosmic radiation detector in saline environment will have all the electromagnetic sensors adjusted at this working frequency (187.5 MHz). Therefore, in formula 1, we will take the *n_sb_* = 1. Thus, the maximum number of the electromagnetic sensors for a detector with the volume 125,000,000 m^3^ and surface dimension of 250,000 m^2^ is 32,000.

Considering the extremely high number of required electromagnetic sensors in order to obtain a cosmic radiation detector in saline environment, it is easy to see that the realization of this system implies very high costs, which are difficult to put up with. Therefore, it is necessary to minimize the cost of realize of the detector for cosmic radiation in saline environment.

## 3. Methodology

The minimization of the development and operation costs for a cosmic radiation detector in the environments in which it can be realized Cherenkov detector, involves finding a technical solution to minimize the number of reception electromagnetic sensors. The technical solution is a new arrangement of the reception electromagnetic sensors inside the volume of an electromagnetic field generated by the Cherenkov cone. This new arrangement of the reception electromagnetic sensors will be chosen so that we can determine the Cherenkov cone using a dedicated software. This software will be implemented in the future. For this, it is necessary to create a database with information about the dielectric parameters of that environment for the two measuring planes (vertically and horizontally). Measurements in the vertical plane will be made in several points, but at the same level up to a horizontal plane as a reference [27] and those for the horizontal plane will be made for positions defined as cardinal points [28].

The practical measurements used in this article were performed in a saline environment. To make the measurements in the salt block, of the dielectric parameters of saline environment, we designed and executed a system consisting in an emission element, a reception electromagnetic sensor, an Anritsu MS2690A signal analyzer, and two adaptive circuits with the cables for links (CDF400, RG58LL). The measurement system is inserted into the salt block in the horizontal and vertical plane [29,30].

The measurements for the vertical plane were performed at three points placed, in the vertical plane, parallel to the floor, at a distance of approximately 1.5 m from the floor (considered as a reference, level zero). The emission element was inserted in turn at two points, diametrically opposed to the position in which the receiving electromagnetic sensor was inserted [27].

**Figure 2:**
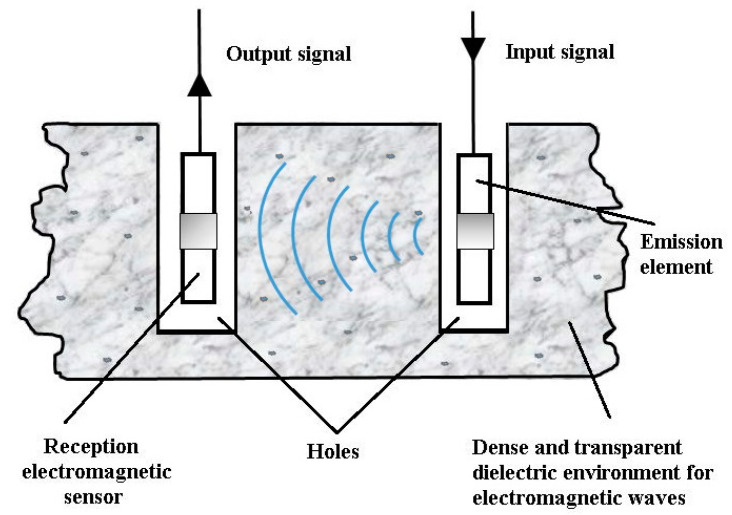
The measurement system inserted into the salt block (horizontal or vertical plane).

Figure 2 shows the introduction of the emission element and the receiving electromagnetic sensor into the environment in which a Cherenkov detector can be made.

To perform the measurements of the dielectric parameters of the saline environment in the horizontal plane, 8 holes (measurement points) were made placed vertically at a depth of about 1 m and arranged in the order of the cardinal points. The receiving electromagnetic sensor was inserted at the center point and was maintained throughout the measurements. A point at 2.04 m from the central point was established in a northerly direction; two points were established in an easterly direction, the first at 5.96 m and the second at 7.98 m from the central point; two points were established in a south direction, the first at 10 m and the second at 12.01 m from the central point; two points were set in a west direction, the first at 9.94 m and the second at 11.94 m from the central point. Thus, it was possible to determine, by subsequent calculations, the dielectric parameters of the saline environment. With these calculated parameters (for both planes—horizontal and vertical) a database will be created, which will contain information about the dielectric parameters of the saline environment (a dielectric map for the saline environment, where the measurements were made) [31]. To determine the Cherenkov cone, which can be generated in this type of environment, we need a software program that can generate a virtual image of the Cherenkov cone in this environment. The virtual image created by the software will simulate very well the real image of the Cherenkov cone in this type of environment [28].

### 3.1. Description of the Equipment Used

Figure 2 shows the introduction of the emission element and the receiving electromagnetic sensor, in the saline environment and this method is used for measurements in the horizontal and vertical planes. This system comprises an emission element (in λ/2) with the adaptive and symmetrizer system included in the space between the two arms of the emission element, a reception electromagnetic sensor, operating on the same frequency as the emission element (187.5 MHz) and the same circuits, coaxial cables (50 Ω) connecting the emission element and the reception electromagnetic sensor, together with impedance adapters “in *Π*” (two impedance adapters “in *Π*”), the connecting cables (50 Ω) between the adapters “in *Π*” and the signal analyzer (with generator included) Anritsu MS2690A.

The emission element is connected via the elements and devices described above to the generator side of the Anritsu MS2690A analyzer, and the reception electromagnetic sensor it is connected via the same elements and devices as the emission element, to the analyzer part of the Anritsu MS2690A analyzer.

The emission element is made of copper pipe with diameter “*d* $\ll L_{e}$” and are calculated for the frequency of 187.5 MHz [32,33] using the formula:

(2) $$L_{e}=L-l=\frac{c}{f\sqrt{\varepsilon_{r}}}$$

where:

$L_{e}$ represents the emission element length in λ/2,
*f* represents the resonance frequency of the emission element [Hz],
$\varepsilon_{r}=5.981+j0.0835$, the real part was taken into account $ℜ\left(\varepsilon_{r}\right)≅6$ because the imaginary part is very small and does not significantly influence the emission element length, and *c* = 3 × 10^8^ m/s represents the speed of light in vacuum space.

**Figure 3:**
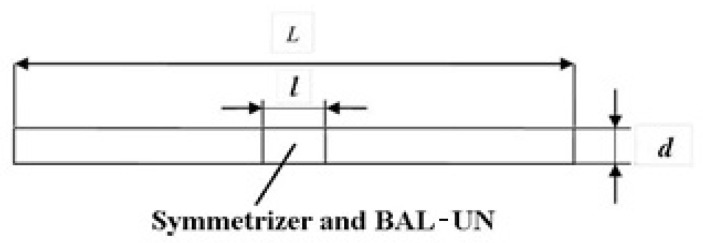
The dimensions of the emission element.

The emission element calculated with the above formula is shown in Figure 3.

Between the two arms of the emission element is mounted an insulator in having the rear a slot in which the symmetrizer and the adaptive system is introduced which is actually a BAL-UN (Balanced-Unbalanced).

We will further analyze all the components of the measurement system to work in a saline environment.

To realize the “BAL-UN” device, we will consider the emission element impedance (*Za*).

The using the impedance adapter “in *Π*”, the imaginary part of the emission element impedance $X_{a}=0$, will be canceled, so we will only discuss the real part of the emission element impedance ($R_{a}$).

Since the emission element is designed to work in saline environment ($ℜ\left(\varepsilon_{r}\right)≅6$) and is in λ/2, then the radiation resistance of the emission element is:

(3) Ra salt=2π3εr saltZ0(Le2λ)2

where $Z_{0}=377\;\Omega$ and λ=cf the wavelength in the air.

Then, $R_{a}=14.266\;\Omega$.

The number of turns in primary and secondary of the “BALUN”, is dependent on the input and output impedances with which it works.

**Figure 4:**
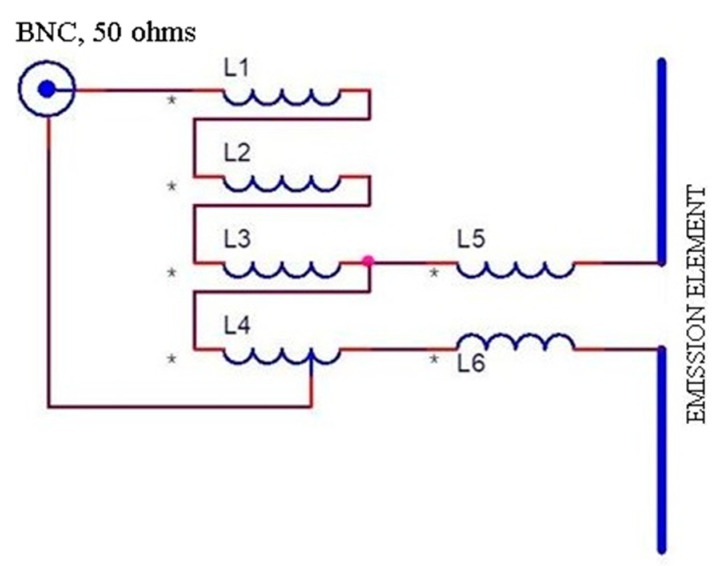
Electrical diagram of the BAL-UN and the symmetrizer (* represents the beginning of the inductor winding).

Figure 4 shows the electrical diagram of the BAL-UN (L1 ÷ L4) and the symmetrizer (L5, L6). Points marked with * are the beginning of the windings.

So, k = 1.872. Then the number of turns for each inductance is *n* = 2 with AWG38 (L1 = L2 = L3 = L4 = L5 = L6). The inductor L4 has an outlet at turn 1.5 from the point marked with *. These inductors will be wound on a toroid with an outer diameter of 6 mm and an inner diameter of 4 mm. For (L1 ÷ L4) 4 coilers were wound in parallel on one of the halves of the torus and on the other two coils (L5, L6) were wound in parallel [34].

**Figure 5:**
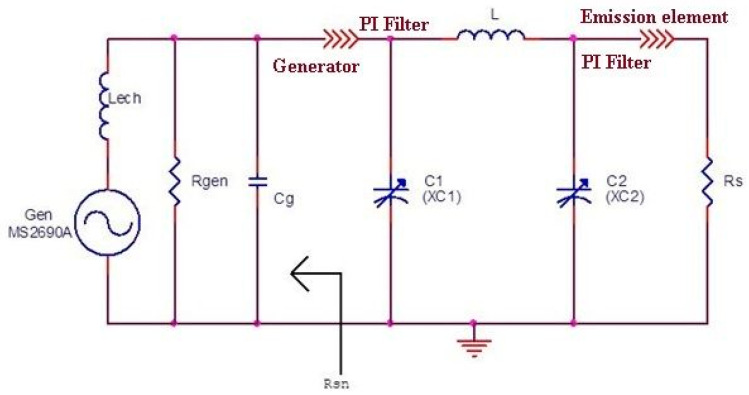
Electrical diagram of the adapter “in *Π*”.

The adapters “in *Π*” are used to cancel the imaginary (reactive) part of the emission element impedance. I used the diagram shown in Figure 5.

The calculation formulas for the adapter “in *Π*” are as follows:

(4) $$X_{2}=\frac{R_{s}}{\sqrt{\frac{R_{s}}{R_{sn}}\left(1+Q^{2}\right)-1}}=XC2$$

(5) $$X_{L}=\frac{QR_{sn}}{1+Q^{2}}\left[1+\frac{1}{Q}\sqrt{\frac{R_{s}}{R_{sn}}\left(1+Q^{2}\right)-1}\right]$$

(6) $$X_{1}=\frac{R_{sn}X_{G}}{QX_{G}-R_{sn}}=XC1$$

(7) Q=RsnωLech

(8) kXG=1ωCg

where:

*R_s_* represents the load resistance of the adapter “in *Π*” (Rs= (7.54)2×Ra=50.154 Ω);
*R_sn_* represents the resistance we want to be at the output of the adapter;
*R_sn_* = 50 Ω (resistance seen by Anritsu MS2690A analyzer—*R_gen_*);
*Q* represents the load quality factor of the adapter “in *Π*”;
  *ω* represents the pulsation at the frequency 187.5 MHz;
*L_ech_* represents the inductance seen at the terminals of the Anritsu MS2690A analyzer for the working frequency of 187.5 MHz;
*C_g_* represents the input-output electrical capacity of the Anritsu MS2690A analyzer.

The Equations (4)–(8) are represented by the adapter diagram “in *Π*” shown in Figure 5.

In the diagram in Figure 5: XC1 = X_1_, XC2 = X_2_ and *X_L_* represents the impedance of the inductor *L*.

For the practical realization of the inductor *L* the formula was used:

(9) L0=200·l[ln(4ld)−0,75]·10−9 [H]

where $L_{0}$ is the inductance determined (*L*) [H], *l* is the length of the line specific to the inductor *L* [m], *d* is the diameter of the line specific to the inductor *L* [m].

**Figure 6:**
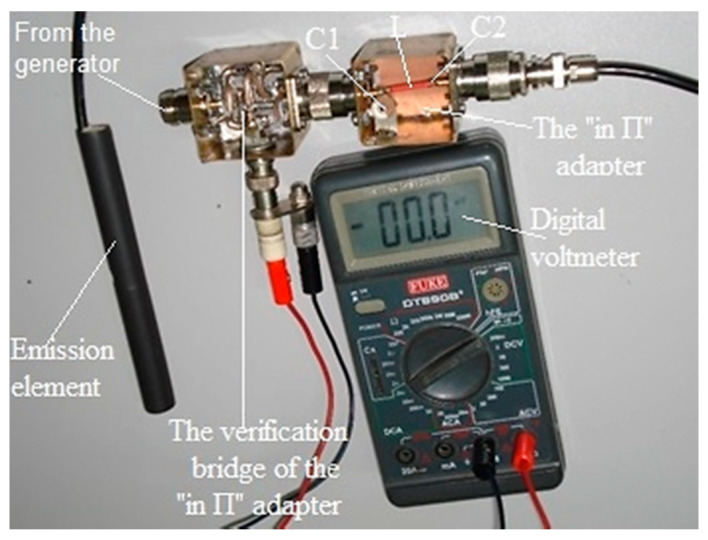
Practical mounting diagram for adjusting the impedance adapter “in *Π*”.

For the adjustment of the impedance adapter “in *Π*” (adjusting the two semi-adjustable capacitor C1 and C2), the mounting scheme shown in Figure 6 was used.

**Figure 7:**
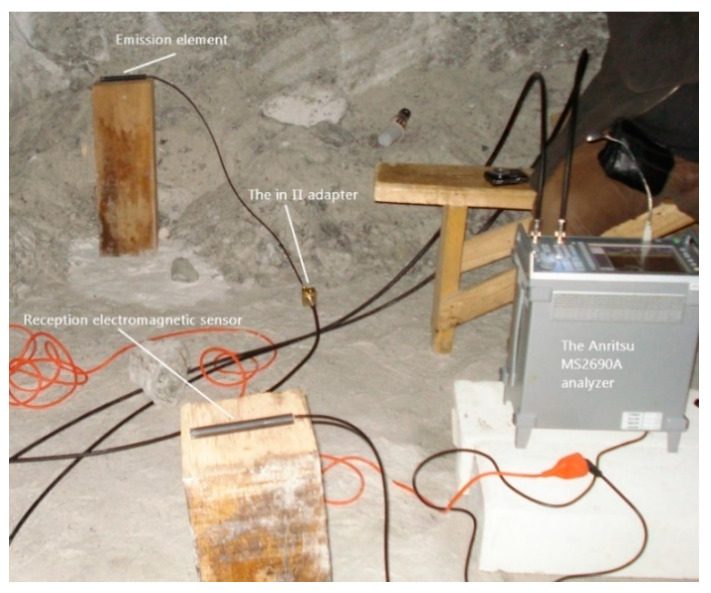
Air measurements.

**Figure 8:**
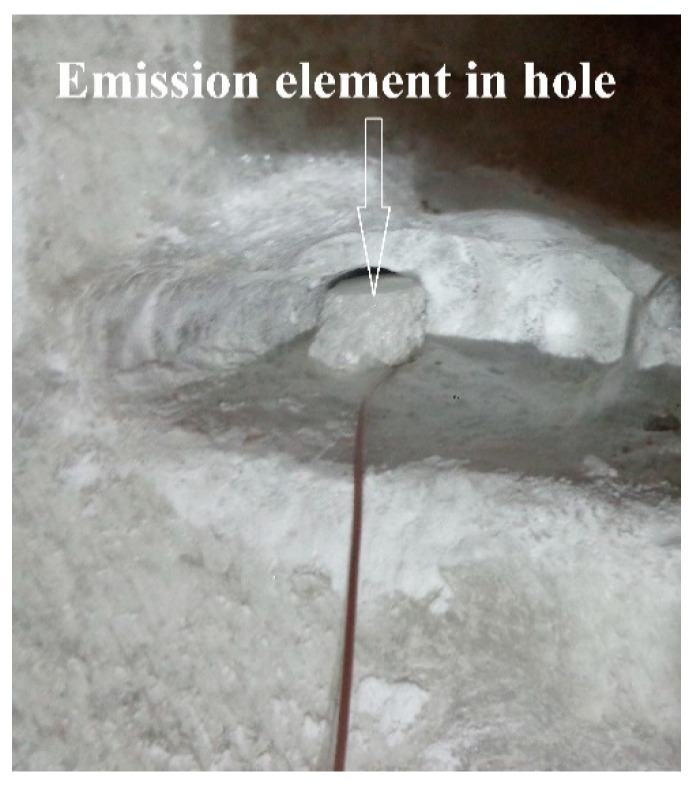
The emission element introduced in saline environment for vertical plane.

**Figure 9:**
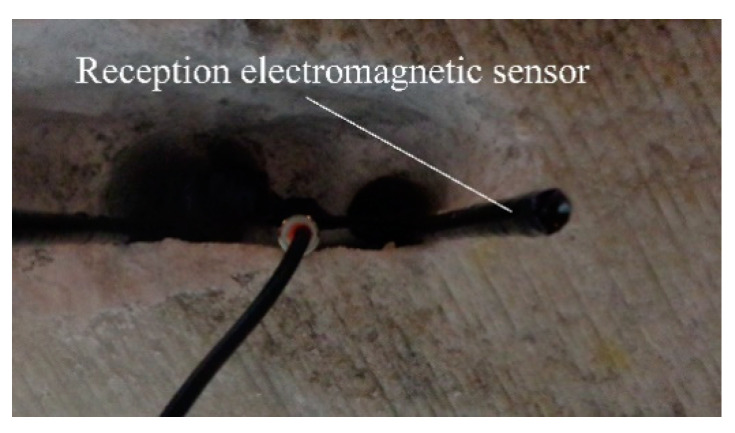
Reception electromagnetic sensor introduced in saline environment for vertical plane.

**Figure 10:**
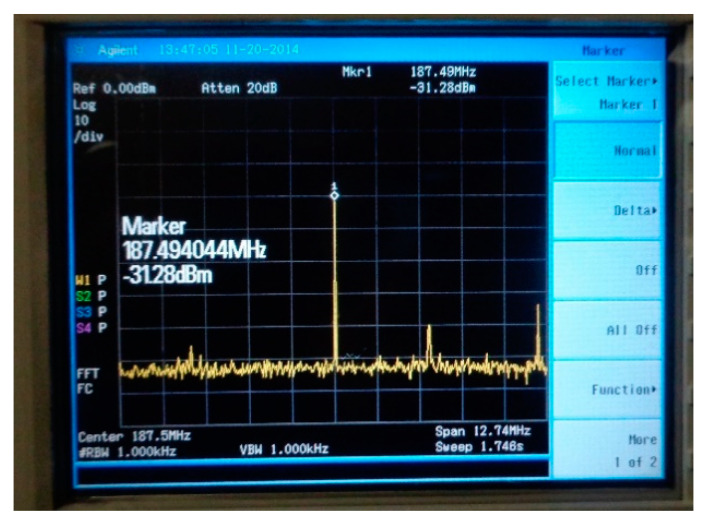
The measurement result for 187.5 MHz frequency for vertical plane.

After the verifications carried out, practical measurements were made inside the mine. Measurements were made with the emission element and reception electromagnetic sensor placed in the air (Figure 7) and with them placed in saline environment for the vertical plane: the emission element introduced in saline environment (Figure 8), reception electromagnetic sensor introduced in saline environment (Figure 9) and the measurement result for 187.5 MHz frequency (Figure 10).

The measurements made in the air helped to eliminate the errors due to parasitic couplings from the package of measurements made in saline environment in the vertical and horizontal planes.

### 3.2. Description of the Physical-Mathematical Phenomenon

In this article, we study the generated Cherenkov cone in any known environment in which the Cherenkov detector is used. The study of cosmic radiation with very high energies (UHE) leads to determine the generation phenomenon of electromagnetic waves. This phenomenon is caused by two different mechanisms by which radio frequency signals are generated. The first mechanism is called geosynchronous radiation. It consists of the generation of radio waves in the terrestrial atmosphere when an interaction between cosmic radiation and the terrestrial atmosphere occurs. These waves are generated by the synchrotron acceleration of electrons and positrons, when they are accelerated by the geomagnetic field [35]. The second mechanism is formed when following the interaction between cosmic radiation and a transparent and dense environment were occurs a surplus of negative charges through the propagation environment. This excess of negative charges generates Cherenkov radiation (the Cherenkov cone is generated). Perpendicular to the side area of this cone, electromagnetic radiation is emitted, which is called the Askaryan effect [36]. In demonstrating the Askaryan effect, numerous experiments were performed in environments, such as ice block, salt and soil of moon. In all these experiments was looked for a frequency of electromagnetic waves (radio frequency) for which we have a coherent effect [37]. The realized studies in determining the Askaryan effect have led to results that conclude that the propagation of radio pulses (electromagnetic radiation in the field of radio frequency is in the form of very short pulses) is dependent on the environment (dense and transparent for radio frequency). Media of propagation of radio pulses by the Askaryan effect are declared unconventional media. As a pioneer in the study of the propagation of radio pulses through unconventional media, was G.A. Askaryan developed the theory of the emission of radio waves in the air due to cosmic radiation since to 1960 and in 1962.

Thus, this theory is applicable in any known environment in which the Cherenkov detector is used. A problem that arises is the study of the propagation of electromagnetic waves through these medium.

Hence the idea of the need to determine an optimal frequency of propagation through saline environment of electromagnetic waves. For this, a study was made on the length of attenuation of electromagnetic waves through the saline environment in the horizontally plane and in the vertically plane. Determining the attenuation length requires a study on the dielectric parameters of the saline environment. All these studies combined with experimental measurements on the saline environment will lead to the determination of the optimization of a Cherenkov detector in the saline environment and the determinate the optimal position for placement the reception electromagnetic sensors.

The propagation of electromagnetic waves is given by Maxwell’s Equations, from which we deduce that the electromagnetic field is an ensemble of two fields (electric and magnetic), variable in time and generated by each other [38].

The intensity of electric field and magnetic field induction satisfy similar equations:

(10) $$\nabla^{2}\vec{E}-\varepsilon \mu \frac{\partial^{2}\vec{E}}{\partial t^{2}}=0$$

(11) $$\nabla^{2}\vec{B}-\varepsilon \mu \frac{\partial^{2}\vec{B}}{\partial t^{2}}=0$$

The electromagnetic waves propagation speed through a medium is deduced from Maxwell’s Equations and is given by:

(12) v=1εμ,

The electromagnetic waves propagation speed in vacuum is: v=1ε0μ0=18,854·10−12·4π·10−7=3·108ms=c

From the measurements made in saline environment it was deduced that *ε_r_* ≈ 6 and *n_D_* = 1.544202 (index of refraction) [37,39].

From here it can be seen that it is very important to determine the dielectric parameters of the saline medium (*ε_r_*) [40]. Thus, we will determine the attenuation length of electromagnetic waves through saline environment. The transport of energy, through a certain medium, generated by the electric and magnetic field depends on the dielectric permittivity and the magnetic permeability of that medium:

(13) $$W_{E}=\frac{\varepsilon {\vec{E}}^{2}}{2};\;W_{B}=\frac{{\vec{B}}^{2}}{2\mu }$$

Considering a region of space, of volume *V* and delimited by the surface *S* and that through this volume an electromagnetic wave propagates, then we can consider the total energy *W* transported by the electromagnetic wave inside this surface:

(14) $$W=\frac{\varepsilon }{2}\underset{V}{\int }{\vec{E}}^{2}dv+\frac{1}{2\mu }\underset{V}{\int }{\vec{B}}^{2}dv$$

Since the energy of the electric and magnetic fields is variable in time, we can determine the expression of the variation in time of the energy of the electromagnetic wave [41]:

(15) $$\frac{\partial W}{\partial t}=-\frac{1}{\mu }\underset{S}{\oint }\vec{P}\cdot d\vec{s}-\underset{V}{\int }\sigma \cdot {\vec{E}}^{2}\cdot dv$$

where $\vec{P}=\vec{E}\times \vec{B}$ represents Poynting’s vector.

From this we deduce that the loss of electromagnetic energy due to propagation through a certain environment is given by the fact that part of the energy will be transported outside the region considered and is represented by the flow of Poynting’s vector (the flow of electromagnetic energy through the closed surface delimits the area; the first integral) and a part of the energy is lost through the Joule effect, in the form of heat (the second integral).

To determine the Cherenkov cone in saline environment it is necessary to know the attenuation length of the saline environment (energy attenuation with propagation length). For this we will start from determining the average rate of energy lost that occurs due to deceleration which is directly proportional to the energy of the particle (*E*). This relation is given by the equation:

(16) dEdx=−1LRE

where: *L_R_* is the radiation length of the environment.

Thus, we can determine the energy *E_x_* which represents the value reached by the particle after traveling the distance x and whose initial energy was *E_0_*. The energy equation after traveling a distance is:

(17) Ex=E0e−xLR

The length of radiation can also be interpreted as the average depth of penetration into the environment at which the average energy decreases exponentially.

Another problem that causes the propagation of electromagnetic waves through a medium is the polarization of the medium, which is in the form of additional electricity field and it occurs in the presence of internal or external electric field. The electric field $\vec{E}$ and electrical polarization $\vec{P}$, represents the two quantities that characterize the state of a dielectric material from an electrical point of view. The sinusoidal variation (ω—pulsation) of the electric field induces a change in the temporary polarization and is also sinusoidal, but with a phase shift (following the electric field). This delay is given by the after-effect [42]

(18) $$E\left(t\right)=E_{0}\sin\left(\omega t\right)$$

(19) $$P\left(t\right)=\varepsilon_{0}\chi_{e}\left(\omega \right)E_{0}\sin\left[\omega t-\varphi \left(\omega \right)\right]$$

where $\varepsilon_{0}=8,854\cdot 10^{-12}$ [F/m], is the permittivity of the vacuum and *χ_e_* = ${\underline{\varepsilon }}_{r}-1$ (${\underline{\varepsilon }}_{r}$ represents the relative dielectric permittivity of the medium) represents the electrical susceptibility and is a dimensionless complex size (χ*_e_* = tensor) [43].

**Figure 11:**
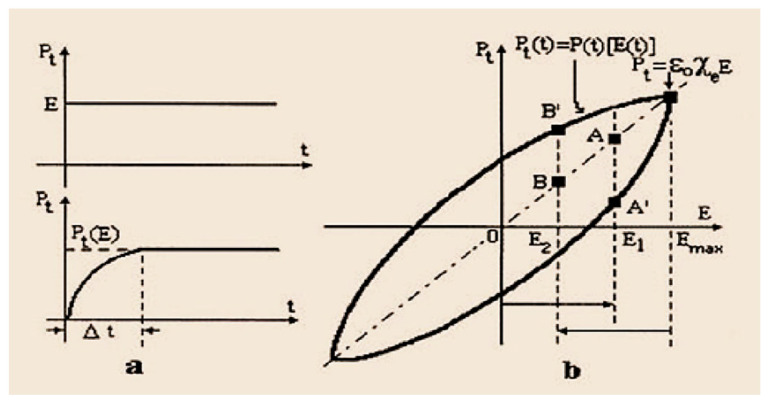
Diagrams associated with the after-effect of the electric field applied to linear dielectrics at: (**a**) sudden variations, (**b**) sinusoidal [42].

Then we can say that at a rapid increase in the electric field: $\Delta E=E_{1}-0$ corresponds to a lower increase of the polarization (Point A in Figure 11b), and to a sudden decrease of the electric field: $\Delta E=E_{max}-E_{2}$ corresponds to a smaller decrease in polarization (Point B’ in Figure 11b).

The salt (NaCl) is a paraelectric medium and is determined by polyatomic substances with asymmetric molecules. It has the susceptibility of high values (varies in inverse relation to temperature), has a post-effect and is dependent on the frequency of the applied alternative electric field. Following the measurements in saline environment, the relative dielectric permittivity of this medium was determined:

(20) $$\varepsilon_{r}=5981+j0.0835$$

The reception electromagnetic sensors of the Cherenkov detector placed in a saline environment capture the information from the electric field as a component part of the electromagnetic waves generated by the Cherenkov cone. Thus, it is necessary to determine the stationary electric field of the saline environment. Thus, the electric field of the density of electric charges distributed (*ρ_v_*) on a volume (*v*) for a medium (*ε_r_*) over which there is no influence of an inner or outer field (the case of a Cherenkov detector placed in a medium, before the interaction with a neutrino) is given by:

(21) $$E=\frac{1}{4\pi \varepsilon_{0}\varepsilon_{r}}{∭}_{V}\rho_{v}\frac{\vec{r}}{r^{3}}dv=0$$

Then we can say that in the case of an imbalance due to the excitation of the environment by an energetic impulse (UHE neutrino) coming from outside the volume (Cherenkov detector) the electric potential (*V_e_*) at a certain point is:

(22) Ve=14πε0εr∭Vρv〈t−→rv〉rdv

where: t−→rv represents the variation variable of the distributed electric charge density (ρv〈t−→rv〉) which for the vertical polarization (*z* axis) are given by $E_{x}\langle z,t\rangle$ and $E_{y}\langle z,t\rangle$ and for the horizontal polarization (*y*-axis) are given by $E_{x}\langle y,t\rangle$ and $E_{z}\langle y,t\rangle$ [44].

The creation of a database with the map of the spatial distribution of the dielectric parameters of the saline environment, will result in the establishment of the locations of the detection elements inside and outside the volume of a Cherenkov detector, based on iterative Equations [29].

Under these conditions we can deduce the iteration relations for the detection of the Cherenkov cone inside the volume of the Cherenkov detector (see relation 23) and outside the volume of the Cherenkov detector (see relation 24).

## 4. Novelties and Expected Results

To minimize the number of the reception electromagnetic sensors of a cosmic radiation detector in any known environment in which the Cherenkov detector is used, we propose:

– the placement points of the reception electromagnetic sensors will be at the points determined by the intersection of the edges of a cube;
– this cube is placed in another cube having the equidistant sides and respects the same rule for placing the reception electromagnetic sensors—being the most convenient solution;
– iteration Equation (Equation (23)) represents the rule for placing cubes in space where: iteration (*i*) (*i*, is equivalent to an increment; initially *i* = 1, for the first application of the Equation (23); to the second application of the Equation (23), *i* = 2; to the third application of the Equation (23), *i* = 3; and so on) determines the length of the edge (*Lc*^3^*_i_*) of the cube; (*p*) represents the iteration pitch and has the same significance as *n_x_*, *n_y_* and *n_z_* from Equation (1); (α) is a constant and it is dependent on the homogeneity of the saline environment; $\alpha ,\;i\in N^{*},\;p\in R\;\mathrm{and}\;p,\;\alpha ,\;i>0$.

If we know the maximum volume of a Cherenkov detector then we can apply the Equation with iteration (Equation (23)) (only inside the volume). For a certain step and after a few iterations, we will generate the entire volume of the detector, at the last iteration. The reception electromagnetic sensors will be placed in the optimal points generated by a software specially designed for this purpose.

(23) $$L_{c_{i}^{3}}=p\left[i+\alpha \left(i-1\right)\right]$$

The determination of the Cherenkov cone can only be possible if it has been generated inside any cube. Thus, for three iterations and for *p* = 25 m, three equidistant cubes will result:

– the first cube will have the $L_{c_{1}^{3}}=25$ m edge; the second cube $L_{c_{2}^{3}}=275$ m and the third cube $L_{c_{3}^{3}}=525$ m. With the three equidistant cubes, we can obtain 32 (it is a suggestive number, it does not represent the number determined by software) the reception electromagnetic sensors compared to the previous situation, where for a cube with the 500 m edge, we obtained 32,000 the reception electromagnetic sensors (much larger number). It can be deduced that the proposed solution, optimizes the Cherenkov detector and contributes to the reduction of realization costs (in the case of our studies—saline environment) of the Cherenkov detector, in any known environment [45]. To optimize the Cherenkov detector (a minimum number of electromagnetic reception sensors) for any known environment in which a Cherenkov detector can be realized, the following steps will be performed:
– the use of a simple device to measure the data collected from to the detection’s elements;
– achievement of the preliminary measurements for the database;
– creation of a database with the map of the spatial distribution of the dielectric parameters of the known environments for the Cherenkov detector;
– placement of the electromagnetic reception sensors in the optimal points determined by the software dedicated for this;
– minimization of the reception electromagnetic sensors number.

Fulfilling the above points makes it possible to make a Cherenkov detector in any possible environment. Neutrinos detected with a Cherenkov detector in saline environment, have much higher energies than in other environments.

The use of reception electromagnetic sensors, in any environment in which a Cherenkov detector can be made, to detect the Cherenkov cone inside the detector volume, represents a new field of their applications, opening a new research direction. The optimization of the Cerenkov detector leads to the minimization and optimal positioning of the receiving electromagnetic sensors. Thus, a patent application was filed on this subject [46].

The method described by this patent application can be applied in any environment known for making a Cherenkov detector. This method is based on creating a database that contains the distribution of the dielectric permittivity of the media in which the Cherenkov detector is made. The necessary measurements for the database are made, by processing the information determined by the measurement results performed in the environments known for the Cherenkov detector (determining the attenuation length of electromagnetic waves, for horizontal and vertical planes, using a reception electromagnetic sensor and an emission element).

The database contains information on the value of the dielectric permittivity of the medium and the Cartesian points assigned to each value. Data entry into the database is done based on dedicated software.

Another variant for determining the position of the points containing information about the dielectric permittivity of the any known environment in which the Cherenkov detector is used, is the polar coordinates (*ρ*, *ϑ*, *φ*) where representation is given by a rotating vector in two planes (it is a more practical variant).

Knowing the values of the dielectric permittivity of the environment, in accordance with the Cartesian (polar) coordinates, it is very easy to determine (software), the optimal points for placing the reception electromagnetic sensors in the entire volume of the Cherenkov detector. This procedure also determines the minimum number of the reception electromagnetic sensors. Thus, through these procedures, a Cherenkov detector is optimized, eliminating the reception electromagnetic sensors whit the same information (eliminating the redundancy of the information).

Extrapolating the ideas presented above, it is clear that the generation of a Cherenkov cone can be detected and, outside the volume of the Cherenkov detector Optimizing the detection of the Cherenkov cone outside the volume of the Cherenkov detector, involves placing a very small number of electromagnetic reception sensors in optimal positions, determined by a dedicated software with the possibility to determine the extrapolation points. This software introduces a minimum number of the reception electromagnetic sensors outside the Cherenkov detector volume and determines their position in the space outside the Cherenkov detector volume.

The detection of the Cherenkov cone outside the volume of the detector [47] is based on the following iteration formula:

(24) $$Lc_{ex}{}_{i}^{3}=p\left[1+i\left(\alpha +1\right)\right]$$

where $Lc_{ex}{}_{i}^{3}$ represents the length of the edge of the surfaces outside the Cherenkov detector volume at one or two iterations; (*p*) the iteration step is the same as in Equation (23); the number of iteration (*i*) is one unit higher than the last iteration for determining the total volume of the Cherenkov detector determined by Equation (23); *α* is a constant; $\alpha ,\;i\in N^{*},\;p\in R\;\mathrm{and}\;p,\;\alpha ,\;i>0$. (Example: *p* = 25 m; *i* = *i*_24_ + 1 = 4; results $Lc_{ex}{}_{i}^{3}=\;625\;m$).

To determine the Cherenkov cone outside the detector volume, but in its immediate vicinity, it is necessary that the energy level determined by the sensors in the planes determined by Equation (24) be lower than the energy level determined by the sensors inside the detector volume.

Then, is determined minimally number of plans in outside the volume of the Cherenkov detector (a plane in each direction: up—down; left—right; front—back).

Then, using these planes in which an optimal (minimum) number of the reception electromagnetic sensors are placed, it is determined (spatially positioned and the electromagnetic energy) of the Cherenkov cone.

Then, using these plans in which an optimal (minimum) number of receiving electromagnetic sensors is placed, the Cherenkov cone is determined (spatially positioned and electromagnetic energy), using a dedicated software and the database with the environment permittivity [48].

## 5. Conclusions

The solution to minimize the number of the reception electromagnetic sensors required to make a Cherenkov detector of cosmic radiation was presented, a general iteration relation that can be applied in any known environment in which the Cherenkov detector is used. By means of this relation, the minimum number of recursive volumes in which can be placed the reception electromagnetic sensors for a given environment can be determined. The optimal placement points of the reception electromagnetic sensors can be determined using a dedicated software.

In this paper an analysis of the optimization of a Cherenkov detector was made and compared with the theoretical analysis of the Cherenkov detector in saline environment (for which we performed the experimental measurements). The maximum number of iterations is depending on the maximum volume desired for making a Cherenkov detector.

In this paper, the focus was on the saline environment but the iteration Equation can be applied for any environment of Cerenkov detector.

We consider it possible to determine the Cherenkov cone, in the vicinity of the volume of the Cherenkov detector.

The reception electromagnetic sensors can be used to determine the dielectric parameters of any environment in which a Cherenkov detector will be made.

## 6. Future Directions to be Approached

The extraction of information carried by neutrinos from cosmic radiation, by detecting the Cherenkov cone in saline and other environments, creates a new approach to the knowledge of the universe [49,50,51].

This paper opens a new research horizon for the detection of the Cherenkov cone outside the detector volume, but in the immediate vicinity, as well as a new field of research of the applications of the reception electromagnetic sensors for the detection of the Cherenkov cone in the other environments.

A new direction for the future is the optimization of Cherenkov detectors in any environment.

The solutions presented in this paper can be applied in any environment [52].

The use of reception electromagnetic sensors for determining the electromagnetic radiation of Cherenkov cone opens a new domain of their application.

## 7. Patent Application

A/00354/12.06.2019, Method for determining the Cherenkov Cone in saline medium outside the volume of the Cherenkov detector”, Authors: Rusu Mădălin Ion, Savu Valeriu and Savastru Dan.

A/00404/07.06.2018, Method of optimization of the Cherenkov detector of electromagnetic radiation in saline environment”, Authors: Savu Valeriu, Rusu Mădălin Ion, Savastru Roxana and Savastru Dan.

## References

[1] Abbasi R., Abdou Y., Abu-Zayyad T., Ackermann M., Adams J., Aguilar J.A., Ahlers M., Altmann D., Andeen K., IceCube Collaboration (2012). An Absence of Neutrinos Associated with Cosmic Ray Acceleration in Gamma-Ray Bursts. Nature.

[2] Watts A. (2012). IceCube Neutrino Observatory Provides New Insights into Origin of Cosmic Rays. https://wattsupwiththat.com/2012/04/20/icecube-neutrino-observatory-provides-new-insights-into-origin-of-cosmic-rays/.

[3] Aguilar J.A. (2013). Icecube Neutrino Results and its Inplications on Astrophysical Models. https://agenda.infn.it/event/5268/contributions/57770/attachments/41492/49291/nutel2013.pdf.

[4] Avrorin A.V., Aynutdinov V.M., Belolaptikov I.A., Bogorodsky D.Y., Budnev N.M., Wischnewski R., Gaponenko O.N., Golubkov K.V., Gress O.A., Gress T.I. (2011). Search for neutrinos from gamma-ray bursts with the Baikal neutrino telescope NT200. Astron. Lett..

[5] Guth A.H. (1998). The Inflationary Universe: The Quest for a NewTheory of CosmicOrigin, Basic Books.

[6] Abbasi R.U., Abu-Zayyad T., Allen M., Amman J.F., Archbold G., Belov K., Belz J.W., Ben Zvi S.Y., Bergman D., Blake S.A. (2008). First Observation of the Greisen-Zatsepin-Kuzmin Suppression. Phys. Rev. Lett..

[7] Castillo-Felisola O., Corral C., Homola P., Zamora-Saa J. (2017). Limits on Beyond Standard Model Messengers as Ultra High Energy Cosmic Rays. arXiv.

[8] Ansoldi S., Antonelli L.A., Arcaro C., Baack D., Babić A., Banerjee B., Bangale P., De Almeida U.B., Barrio J.A., Gonzalez J.B. (2018). The blazar TXS 0506+056 associated with a high-energy neutrino: Insights into extragalactic jets and cosmic ray acceleration. Astrophys. J. Lett..

[9] Abeysekara A.U., Alfaro R., Alvarez C., Arceo R., Arteaga-Velázquez J.C., Rojas D.A., Belmont-Moreno E., BenZvi S.Y., Brisbois C., Capistrán T. (2019). All-sky Measurement of the Anisotropy of Cosmic Rays at 10 TeV and Mapping of the Local Interstellar Magnetic Field. Astrophys. J..

[10] Zhu Q.Y., Wang X.Y. (2016). Probing the environment of gravitational-wave transient sources with TeV afterglow emission. Astrophys. J. Lett..

[11] Dowell J., Taylor G.B. (2018). The Extragalactic Radio Background Below 100 MHz. Astrophys. J. Lett..

[12] Savin A., Steigmann R., Bruma A., Šturm R. (2015). An Electromagnetic Sensor with a Metamaterial Lens for Nondestructive Evaluation of Composite Materials. Sensors.

[13] Marc H.C. (2006). Harvesting Ambient Radio Frequency Electromagnetic Energy for Powering Wireless Electronic Devices, Sensors and Sensor Networks and Applications Thereof.

[14] Gaug M., Fegan S., Mitchell A.M.W., Maccarone M.C., Mineo T., Okumura A. (2019). Using Muon Rings for the Calibration of the Cherenkov Telescope Array: A Systematic Review of the Method and Its Potential Accuracy. Astrophys. J. Suppl. Ser..

[15] Adriani O., Barbarino G.C., Bazilevskaya G.A., Bellotti R., Boezio M., Bogomolov E.A., Bongi M., Bonvicini V., Bruno A., Cafagna F. (2018). Unexpected cyclic behavior in cosmic-ray protons observed by PAMELA at 1 au. Astrophys. J. Lett..

[16] Caravaca J., Descamps F.B., Land B.J., Orebi Gann G.D., Wallig J., Yeh M. (2017). Probing Cherenkov and Scintillation Light Separation for Next-Generation Neutrino Detectors. J. Phys. Conf. Ser..

[17] Pfrommer C., Pakmor R., Simpson C.M., Springel V. (2017). Simulating Gamma-Ray Emission in Star-forming Galaxies. Astrophys. J. Lett..

[18] Gaigalas G., Kato D., Rynkun P., Radžiūtė L., Tanaka M. (2019). Extended Calculations of Energy Levels and Transition Rates of Nd II-IV Ions for Application to Neutron Star Mergers. Astrophys. J. Suppl. Ser..

[19] Abeysekara A.U., Albert A., Alfaro R., Alvarez C., Álvarez J.D., Camacho J.R.A., Arceo R., Arteaga-Velázquez J.C., Arunbabu K.P., Rojas D.A. (2019). Measurement of the Crab Nebula at the Highest Energies with HAWC. Astrophys. J..

[20] Engel R., Sekel D., Stanev T. (2001). Neutrinos from propagation of ultra-high energy protons. Phys. Rev. D.

[21] Yasuda H., Lee S.H. (2019). Time Evolution of Broadband Nonthermal Emission from Supernova Remnants in Different Circumstellar Environments. Astrophys. J..

[22] Carroll B., Ostlie D., Addison P. (2007). Ebook—An Introduction to Modern Astrophysics.

[23] Toshio K., Masami C. (2003). Microwave Properties of Rock Salt and Lime Stone for Detection of Ultra-High Energy Neutrinos. Part. Astrophys. Instrum..

[24] Chiba M., Arakawa Y., Kamijo T., Yabuki F., Yasuda O., Chikashige Y., Ibe K., Kon T., Shimizu Y., Taniuchi Y. (2009). Radar for salt ultra-high-energy neutrino detector and contribution of W-gluon fusion process to collision of neutrinos against protons. Nucl. Instrum. Methods Phys. Res..

[25] Hashimoto F., Ote K., Ota R., Hasegawa T. (2019). A feasibility study on 3D interaction position estimation using deep neural network in Cherenkov-based detector: A Monte Carlo simulation study. Biom. Phy. Eng.Exp..

[26] Zakir K., Muhammad H.M., Rahman S.U., Sajjad M., Lin F., Sun L. (2020). A Single-Fed Multiband Antenna for WLAN and 5G Applications. Sensors.

[27] Savu V., Fratu O., Rusu M.I., Savastru D., Tenciu D., Vulpe A., Craciunescu R. (2018). Determination of the electromagnetic wave propagation for the detection of the Cherenkov radiation cone in salt environment. UPB Sci. Bull. Ser. A.

[28] Savu V., Marghescu I., Fratu O., Halunga S., Badescu A.M. (2013). Antenna design for electromagnetic waves propagation studies through the salt ore. UPB Sci. Bull. Ser. C.

[29] Savu V., Rusu M.I., Savastru D. (2020). Ebook—Nuclear Power Plant.—Chapter—Optimization of Cosmic Radiation Detection in Saline Environment.

[30] Gabriela A., Nikolay A. (2020). Small Antennas for Wearable Sensor Networks: Impact of the Electromagnetic Properties of the Textiles on Antenna Performance. Sensors.

[31] Amer A.-B., Iulia A.M., Norocel C., Pantazica M. (2020). Modified Split Ring Resonators Sensor for Accurate Complex Permittivity Measurements of Solid Dielectrics. Sensors.

[32] Alexandru T., Alina B. (2020). Wideband Dual-Polarized VHF Antenna for Space Observation Applications. Sensors.

[33] Muhammad K.K., Changhyeong L., Heejun P., Sungtek K. (2020). A Fully-Printed CRLH Dual-Band Dipole Antenna Fed by a Compact CRLH Dual-Band Balun. Sensors.

[34] Andreea C., Razvan D.T. (2020). Evaluation and Impact Reduction of Common Mode Currents on Antenna Feeders in Radiation Measurements. Sensors.

[35] McIntyre P., Sattarov A. (2006). Tripling the LHC: The Path from Technology to Discovery. Dark Matter in Astro- and Particle Physics.

[36] Huege T. (2009). Simulations and theory of radio emission from cosmic ray air showers. Nucl. Instrum. Meth. Suppl. A.

[37] Saltzberg D., Gorham P., Walz D., Field C., Iverson R., Odian A., Resch G., Schoessow P., Williams D. (2001). Observation of the Askaryan Effect: Coherent Microwave Cherenkov Emission from Charge Asymmetry in High-Energy Particle Cascades. Phys. Rev. Lett..

[38] Eringen A.C. (1963). On the foundations of electroelastostatics. Int. J. Eng. Sci..

[39] https://pubchem.ncbi.nlm.nih.gov/compound/5234#section=Corrosivity.

[40] Eringen A.C. (1981). On nonlocal plasticity. Int. J. Eng. Sci..

[41] http://www.scritube.com/stiinta/fizica/Undele-electromagnetice-Poluar81593.php.

[42] http://www.scritube.com/stiinta/fizica/Materiale-dielectrice211119722.php.

[43] Smith G.F. (1971). On isotropic functions of symmetric tensors, skew-symmetric tensors and vectors. Int. J. Eng. Sci..

[44] Dunkin J.W., Eringen A.C. (1963). On the propagation of waves in an electromagnetic elastic solid. Int. J. Eng. Sci..

[45] Landoni M., Romano P., Vercellone S., Knödlseder J., Bianco A., Tavecchio F., Corina A. (2019). A Cloud-based Architecture for the Cherenkov Telescope Array Observation Simulations: Optimization, Design, and Results. Astrophys. J. Suppl. Ser..

[46] Savu V., Rusu M.I., Savastru R., Savastru D. (2018). Method for Optimizing Cherenkov Electromagnetic Radiation Detector in Saline Medium.

[47] Rusu M.I., Savu V., Savastru D. (2019). Method of Determining the Cherenkov Cone in Saline Environment Outside the Volume of the Cherenkov Detector.

[48] Zvonimir S., Katarina C., Marko B., Rajo-Iglesias E. (2020). Glide-Symmetric Holey Structures Applied to Waveguide Technology: Design Considerations. Sensors.

[49] Visbal E., McQuinn M. (2018). The Impact of Neutral Intergalactic Gas on Lyα Intensity Mapping during Reionization. Astrophys. J..

[50] Feng C., Holder G. (2018). Enhanced Global Signal of Neutral Hydrogen Due to Excess Radiation at Cosmic Dawn. Astrophys. J..

[51] Draine B.T., Miralda-Escude J. (2018). Absorption by Spinning Dust: A Contaminant for High-redshift 21 cm Observations. Astrophys. J..

[52] Fleischhack H. (2015). A template method for measuring the iron spectrum in cosmic rays with Cherenkov telescopes. J. Phys. Conf. Ser..

